# Intraoperative patellofemoral load varies between total knee replacement and patellofemoral joint resurfacing, and across TKR implant systems

**DOI:** 10.1007/s00402-026-06500-3

**Published:** 2026-09-09

**Authors:** Louis Phillipson, John Naybour, Annabel Watson, Nick London, David Barrett

**Affiliations:** 1 Eventum Orthopaedics, Leeds, UK; 2 Leeds Beckett University, Leeds, UK; 3 School of Engineering and Sciences, University of Southampton, Southampton, UK

**Keywords:** Quadsense, Patellofemoral joint, Total knee replacement, Implant design, Intraoperative load measurement

## Abstract

**Introduction:**

Patellofemoral joint loading is influenced by implant design and surgical execution during knee replacement. Altered patellofemoral mechanics have been implicated in postoperative symptoms, yet the magnitude and variability of intraoperative load changes across different replacement procedures and implant systems remain incompletely characterised. The aim of this registry study was to describe and quantify intraoperative patellofemoral joint load changes following total knee replacement (TKR) and patellofemoral joint resurfacing (PFJR), focusing on cumulative and compartment-specific load redistribution.

**Materials and methods:**

This observational registry study analysed prospectively collected intraoperative patellofemoral joint load measurements from 291 knee replacement procedures. Load was recorded in medial, lateral, superior, and inferior compartments before and after trial component implantation. Absolute and percentage changes in compartmental and cumulative load were calculated. Descriptive analyses examined the distribution and variability of load change across procedure type and implant system, with implant comparisons restricted to TKR procedures. No formal hypothesis testing was pre-specified, and no adjustment for potential confounding variables was undertaken.

**Results:**

Cumulative patellofemoral load change demonstrated substantial inter-individual variability. While most cases clustered around small percentage changes, a distinct subset of patients exhibited large cumulative load increases exceeding + 70%. TKR procedures demonstrated a broader distribution and higher observed frequency of large cumulative load increases compared with PFJR. Implant-specific analysis of TKR procedures revealed descriptive differences in cumulative load distributions between implant cohorts, with certain implant systems showing a higher observed frequency of large load increases within this dataset. Compartmental magnitude analysis demonstrated that lateral and superior compartments accounted for most of the load redistribution, whereas medial and inferior compartments contributed minimally.

**Conclusions:**

Intraoperative patellofemoral load redistribution following knee replacement is highly variable and was observed to differ descriptively between procedure types and implant systems. Load changes were predominantly observed in the lateral and superior compartments, with a meaningful subset of patients experiencing large cumulative increases. Registry-based intraoperative load measurement allows detailed characterisation of patellofemoral biomechanics beyond cumulative metrics alone and may inform future investigations into implant design and surgical optimisation. Given the observational registry design and absence of confounder adjustment, these findings should be interpreted as descriptive associations rather than causal relationships.

## Introduction

Isolated compartment arthrosis of the knee presents a complex challenge in knee replacement surgery, particularly when the patellofemoral joint is involved. Patellofemoral pain and dysfunction remain common causes of patient dissatisfaction following knee replacement, even when tibiofemoral components are otherwise well functioning. Altered patellofemoral joint loading has been proposed as a potential contributor to these symptoms, yet the biomechanical consequences of different knee replacement procedures on patellofemoral loading remain incompletely understood.

Total knee replacement alters knee kinematics through changes in alignment, component geometry, and soft tissue balance. These changes may redistribute load across the patellofemoral joint, with potential implications for pain, function, and implant longevity. Patellofemoral joint resurfacing procedures aim to address isolated disease while preserving native tibiofemoral compartments and may therefore produce different patterns of patellofemoral load redistribution. However, existing studies have often focused on mean changes or single surgical techniques, with limited attention to inter-individual variability or directional patterns of load redistribution.

Recent advances in intraoperative load measurement technologies allow direct quantification of patellofemoral joint loading during surgery. These measurements provide an opportunity to characterise real-time biomechanical changes associated with implant placement and surgical technique in routine clinical practice. To date, most published studies have been limited by small sample sizes or experimental conditions, and large registry-based analyses describing patellofemoral load behaviour across procedures, implants, and alignment strategies remain scarce.

The purpose of this study was to analyse intraoperative patellofemoral joint load changes within a large, multi-centre registry of knee replacement procedures. Specifically, the study aimed to quantify the magnitude and variability of cumulative and compartment-specific load changes, to describe observed differences in load redistribution between TKR and PFJR procedures, and to characterise directional patterns observed in association with implant choice. By focusing on variability and directional behaviour rather than mean values alone, this study seeks to provide a more detailed understanding of patellofemoral load redistribution during knee replacement.

## Methods

### Study design

This study was an observational analysis of prospectively collected anonymised registry data. Intraoperative patellofemoral joint load measurements were recorded during routine knee replacement procedures and analysed retrospectively. Data collection formed part of routine post-market clinical follow-up activities for an intraoperative patellofemoral load measurement device and did not alter standard surgical practice. All data were fully anonymised prior to analysis. In accordance with local governance arrangements for anonymised post-market registry data, formal ethical approval and individual patient consent were not required.

### Study setting and period

The registry included procedures performed between July 2023 and December 2025 across 17 hospitals. Data were contributed by 23 consultant orthopaedic surgeons, all of whom were experienced in knee replacement and trained in the use of the patellofemoral joint load measurement system (Quadsense System, Eventum Orthopaedics).

### Participants and eligibility

All primary knee replacement procedures in which the patellofemoral joint load measurement device was used were eligible for inclusion. Procedures comprised primary total knee replacement (TKR) and patellofemoral joint resurfacing (PFJR). Eligible procedures were required to include patellar resurfacing and to have two complete intraoperative measurement captures: (1) a “native” measurement recorded following patellar resection and prior to implantation of femoral, tibial, or trochlear components, and (2) a post-implant “trial component” measurement recorded with trial components in situ (resurfaced patella with trial femoral components, and trial tibial components in TKR procedures). Each capture comprised four sensor readings (medial, lateral, superior, inferior); therefore, a complete case required eight recorded sensor values per procedure (four native + four trial), all of which had to be present (recorded values of 0 were accepted as valid).

A total of 296 procedures were identified within the registry during the study period. Two cases were excluded prior to analysis due to unsuitable procedure type (secondary knee procedures), and three were excluded due to anomalous load recordings consistent with measurement error or device misuse, leaving 291 procedures for analysis. Use of the device requires patellar resurfacing and is not indicated for revision surgery; revision procedures were therefore not included. Although the device is formally indicated for use in total knee replacement, some participating surgeons elected to use the device during PFJR procedures.

Procedure type was explicitly recorded in 251 of 291 cases (TKR *n* = 223; PFJR *n* = 28). In 40 cases, the procedure-type field was incomplete in the registry dataset. The PFJ load measurement device is formally indicated for use in primary total knee replacement. However, registry data included procedures recorded by participating surgeons in which the device was also used during PFJR procedures. As all recorded cases involved either TKR or PFJR procedures, cases with missing procedure-type coding were known clinically to represent one of these two procedure types. These cases were included in overall analyses but excluded from stratified comparisons between TKR and PFJR.

### Patellofemoral joint load measurement

Patellofemoral joint load was measured intraoperatively using the Quadsense System (Eventum Orthopaedics), a force measurement device indicated for use during primary total knee replacement. The device has undergone verification, validation, calibration, and repeatability testing required for clearance for clinical use in the United Kingdom, New Zealand, and the United States.

The sensor device, Quadsense, is positioned on the posterior surface of the resected patella, with the sensing surface facing the femoral trial component during knee flexion and extension. The device is positioned with the sensor apex aligned with the intended apex of the patellar implant. The device contains four sensing quadrants corresponding to the superior, inferior, medial, and lateral regions of the patellar articular surface. During placement, the superior sensor is oriented superiorly, the inferior sensor inferiorly, and the medial and lateral sensors positioned accordingly relative to the patellar anatomy. This orientation is maintained for both left and right knees, resulting in a mirror configuration between limbs. For the purposes of analysis, data from left and right knees were combined by anatomical direction, such that medial and lateral measurements were grouped together regardless of limb laterality.

Load measurements were obtained immediately following patellar resection and prior to femoral, tibial, or trochlear bone resections. A second set of measurements was recorded with trial components in situ. During each measurement, the limb was non-load bearing with the patient supine and quadriceps musculature relaxed under anaesthesia. Surgeons manually cycled the knee through extension and flexion.

The Quadsense System is indicated for use during primary total knee replacement. Data included in this registry were collected as part of routine post-market clinical follow-up activities. In some cases, participating surgeons elected to use the device during PFJR as part of their intraoperative assessment. Such use represents surgeon-directed use outside the device’s labelled indication, and participating surgeons were aware that the device is formally indicated for primary total knee replacement procedures. These cases were recorded in the registry dataset and included in the present analysis for descriptive comparison. No study-specific protocol required device use outside its indicated application.

### Measurement acquisition protocol

Each valid measurement required a minimum of three consistent flexion–extension cycles recorded over a maximum duration of 12 s. The reported compartmental load value represents the mean of the peak maxima identified across at least three consistent cycles. Cycles were averaged to derive a single representative load value per compartment for each measurement state (native and trial).

### Peak detection and signal processing

Peak values were defined as local maxima bounded by adjacent local minima within each flexion–extension cycle. Small transient peaks were excluded if their area-under-the-curve was less than 1% of the largest detected peak within that recording period. A lower rejection threshold of 1 N was applied, with values below this level not recorded. Automatic signal smoothing and noise reduction were applied by the device software prior to peak extraction. Surgeons were provided with real-time graphical feedback (force versus time) and were able to repeat measurements if traces appeared visually inconsistent.

### Flexion angle and standardisation

Flexion angle was not formally standardised or recorded by the device. Surgeons were instructed to perform similar ranges and cycling behaviour between native and trial measurements within each case to maximise intra-case comparability. The study therefore emphasises within-case comparative consistency (native vs. trial) rather than between-case standardisation of absolute flexion angles.

### Sensor calibration and drift

Sensors are single-use and calibrated prior to sterilisation and packaging. No intraoperative recalibration is required. As each sensor is used only once, drift over time is not applicable.

### Surgical technique considerations

To minimise disruption to routine surgical workflow, operative technique (including femoral component rotation strategy, lateral release, and tourniquet use) was not standardised or mandated by the study protocol and was not routinely recorded. Patellar resection was performed according to the surgeon’s standard technique but was required to accommodate the sensor while maintaining adequate patellar thickness. While sensor placement on the resected patella was not formally standardised across surgeons, instructions for use recommend positioning consistent with the intended patellar implant apex. Consistency of sensor placement and cycling technique within each case was emphasised to optimise comparison between native and trial measurements.

### Load variables and definitions

Force readings were recorded from four sensor quadrants corresponding to the anatomical medial, lateral, superior, and inferior regions of the resected patella. Cumulative patellofemoral joint load was defined as the sum of the force readings from all four compartments. Absolute and percentage changes in compartmental and cumulative load were calculated between pre-implantation and trial component measurements.

For compartmental analyses, directional behaviour was summarised by categorising each compartmental load change according to the sign of change between native and trial measurements (increase > 0; decrease < 0; exact equality = 0). No tolerance band or percentage threshold was applied. Magnitude-based analyses were performed using the modulus of absolute change to describe the relative contribution of each compartment to overall load redistribution, independent of direction.

### Outcome measures

Percentage change in cumulative patellofemoral joint load was used extensively as a descriptive outcome measure in frequency-based analyses. Secondary outcomes included absolute and percentage changes in compartment-specific load and the relative contribution of each anatomical compartment to total load redistribution. Analyses describing load patterns were stratified by procedure type and implant system (within TKR procedures).

### Frequency histogram binning

Frequency histograms were constructed using 10% bin widths. To improve visual resolution across most observations while preserving extreme values, outer bins were defined at < − 70% and > + 70%. This threshold was selected following inspection of the full dataset, which demonstrated that approximately the upper decile of cumulative load changes exceeded + 70%. Values beyond these limits were retained and grouped within the outer bins rather than excluded from analysis. This binning strategy was used for visualisation purposes only and did not alter underlying continuous data analyses.

### Surgical variables

Surgical variables recorded prospectively included procedure type and implant system (Attune, Triathlon, Persona, Omni, Conformis). Analyses comparing implant systems were restricted to TKR procedures. Implant-stratified analyses were limited to implant systems with sufficient sample size to permit meaningful descriptive comparison. Specifically, Attune (*n* = 132), Persona (*n* = 26), and Triathlon (*n* = 53) cohorts were included. Conformis (*n* = 2) and Omni (*n* = 5) cohorts were excluded from implant-level comparison due to limited sample size. No multivariable adjustment for patient-, surgeon-, or centre-level factors was performed. Implant selection was determined by surgeon preference and was not randomised. Implant use was strongly clustered by surgeon and site within the registry dataset.

### Statistical analysis

Analyses focused on describing variability, distribution, and compartmental patterns of patellofemoral joint load change. Data processing, descriptive analyses, and figure generation were performed using Microsoft Excel (Microsoft Corporation, Redmond, WA, USA).

The author had full access to the anonymised registry dataset used in this study. Data were exported directly from the registry database and analysed in their entirety according to predefined inclusion criteria.

Missing data were handled using complete-case analysis for variables required for each specific analysis. Load measurement data were complete for all included cases due to the eligibility criteria requiring eight valid sensor readings per procedure. Cases with missing procedure-type coding were included in overall descriptive analyses but excluded from stratified comparisons between TKR and PFJR procedures. Implant type was missing in five TKR cases and these were excluded from implant-level comparisons. No imputation of missing values was performed.

This study was exploratory and descriptive in nature. All eligible cases within the registry dataset were included in the analysis to minimise selection bias. No formal hypothesis testing was pre-specified, and no inferential statistical testing was undertaken. Results are therefore presented descriptively and interpreted in the context of an exploratory observational analysis.

Where possible, analyses were stratified to explore associations between procedure type and implant system. However, due to the observational nature of the registry and real-world surgical practice, complete isolation of all variables was not feasible. Comparisons presented in this study should therefore be interpreted as descriptive associations within the available dataset rather than isolated effects of any single variable.

## Results

### Cohort overview

A total of 296 registry cases were screened during the study period. Two cases were excluded prior to analysis due to unsuitable procedure type (secondary knee procedures), and three were excluded due to anomalous load recordings consistent with measurement error or device misuse, leaving 291 procedures for analysis.

Procedure type was recorded in 251 cases (TKR *n* = 223; PFJR *n* = 28). The remaining 40 cases had incomplete procedure-type coding within the registry dataset and were included in overall analyses but excluded from procedure-type stratified comparisons.

**Fig. 1:**
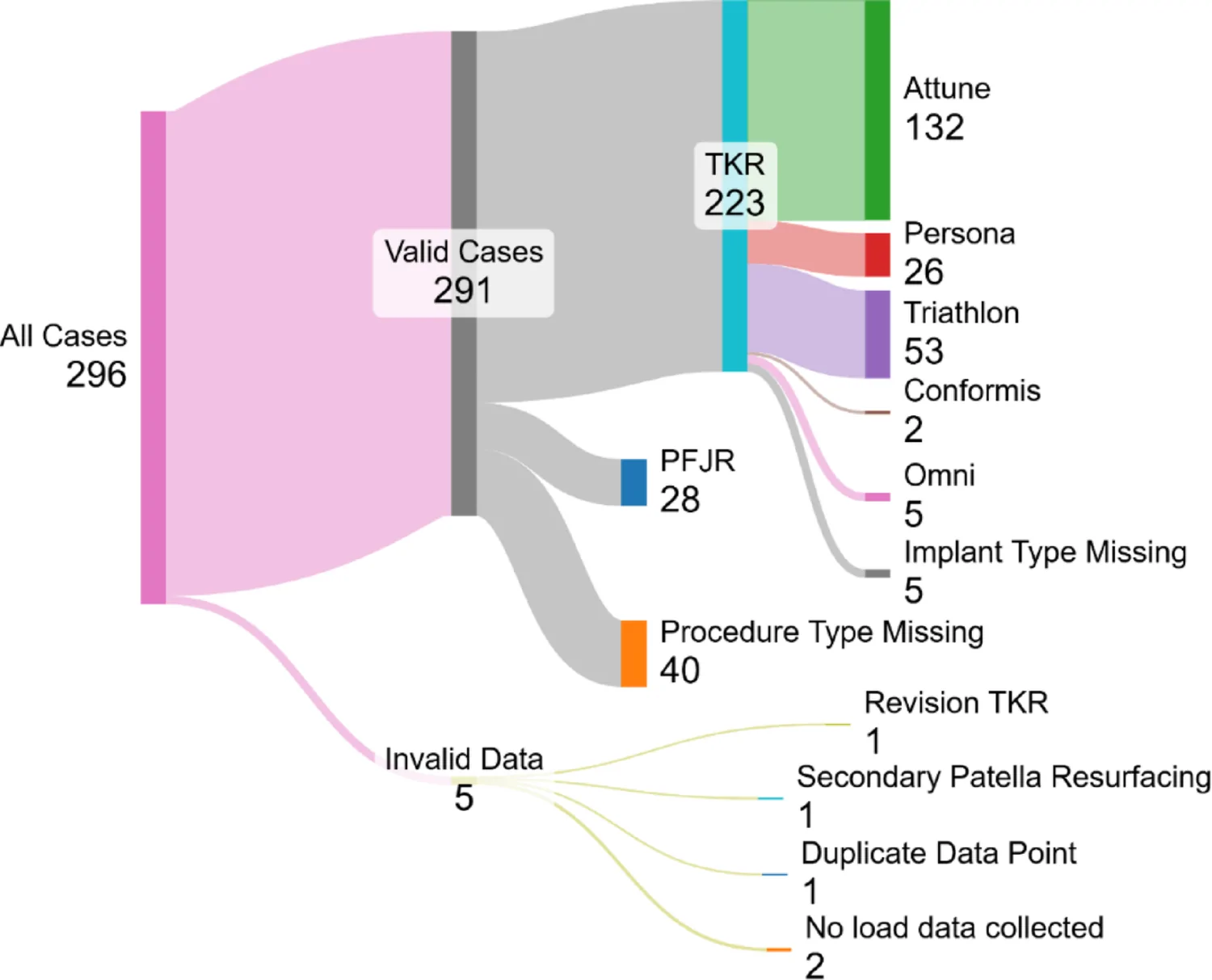
Diagram of cohort derivation

Procedures were performed across multiple centres using a range of implant systems. All analyses relate to intraoperative patellofemoral joint load measurements recorded before and after implantation (Fig. 1).

### Overall cumulative patellofemoral load change

**Fig. 2:**
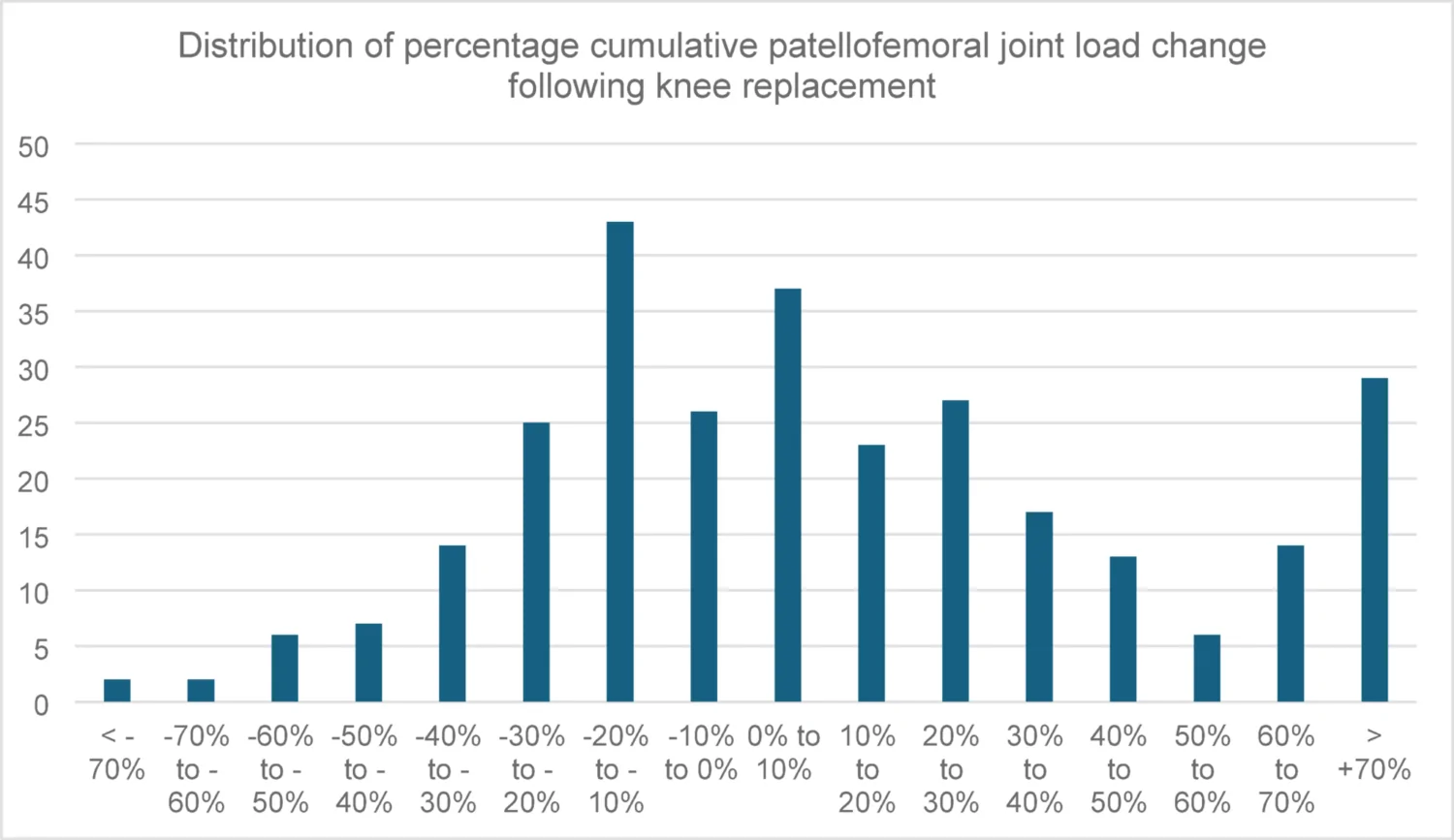
Distribution of percentage cumulative patellofemoral joint load change following all knee replacement procedures (*n* = 291). Histograms use 10% bin widths with outer bins defined at < − 70% and > + 70%, corresponding to the upper decile of cumulative load changes of all cases and an equivalent negative threshold

Figure 2 shows the distribution of percentage change in cumulative patellofemoral joint load across all included cases. The distribution demonstrated substantial inter-individual variability. While most cases clustered around relatively small percentage changes, a distinct subgroup of cases fell within the upper > + 70% bin, representing the upper decile of cumulative load increases across the cohort. Reductions in cumulative load were observed less frequently and were generally of smaller magnitude.

Across all cases, the median cumulative percentage change in patellofemoral joint load was + 3.79% (interquartile range − 15.44% to + 34.08%).

### Effect of procedure type

**Fig. 3:**
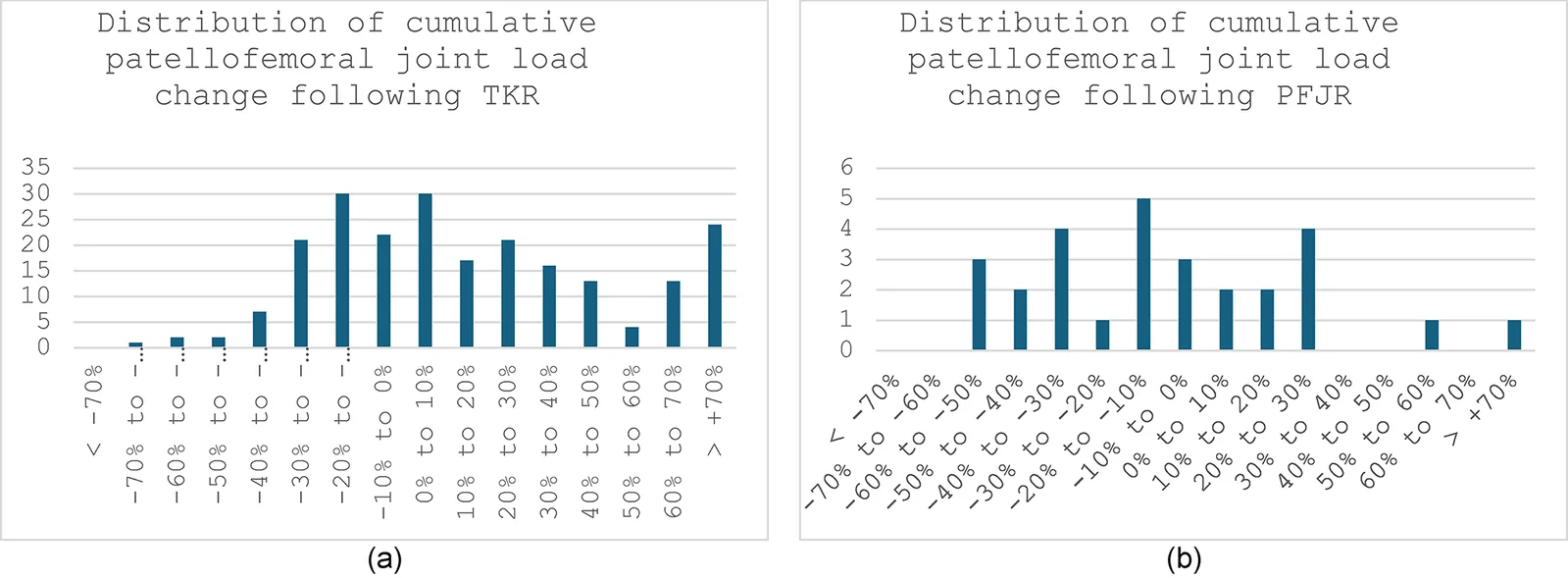
Distribution of percentage cumulative patellofemoral joint load change following **a** TKR (*n* = 223) and **b** PFJR (*n* = 28) procedures. 10% bin widths are used, with outer bins of <−70% and > + 70%, corresponding to the outer limits derived from the upper decile of the all cases cohort (*n* = 291)

When stratified by procedure type (Fig. 3), differences in the distribution of cumulative load change were observed between TKR and PFJR procedures. TKR cases were observed to demonstrate a broader distribution of percentage change, with a higher observed frequency of large positive cumulative load increases. In contrast, PFJR cases clustered more closely around 0% change, with fewer instances of extreme load increase and a slight tendency towards small negative changes.

For TKR cases, the median cumulative percentage change in patellofemoral joint load was + 9.09% (interquartile range − 11.17% to + 38.66%). For PFJR cases, the median cumulative percentage change in patellofemoral joint load was − 11.44% (interquartile range − 34.64% to + 14.47%).

### Effect of implant choice in TKR

To describe patterns according to implant selection, analyses were restricted to TKR procedures with recorded implant data (*n* = 223). Implant-stratified analyses were performed for Attune (*n* = 132), Persona (*n* = 26), and Triathlon (*n* = 53) cohorts. Conformis (*n* = 2) and Omni (*n* = 5) cases were excluded from implant-level comparison due to limited sample size.

For TKR cases using Attune, the median cumulative percentage change in patellofemoral joint load was + 3.34% (interquartile range − 12.61% to + 28.92%). For TKR cases using Persona, the median cumulative percentage change in patellofemoral joint load was − 0.38% (interquartile range − 19.14% to + 33.31%). For TKR cases using Triathlon, the median cumulative percentage change in patellofemoral joint load was − 10.23% (interquartile range − 15.04% to + 56.27%).

**Fig. 4:**
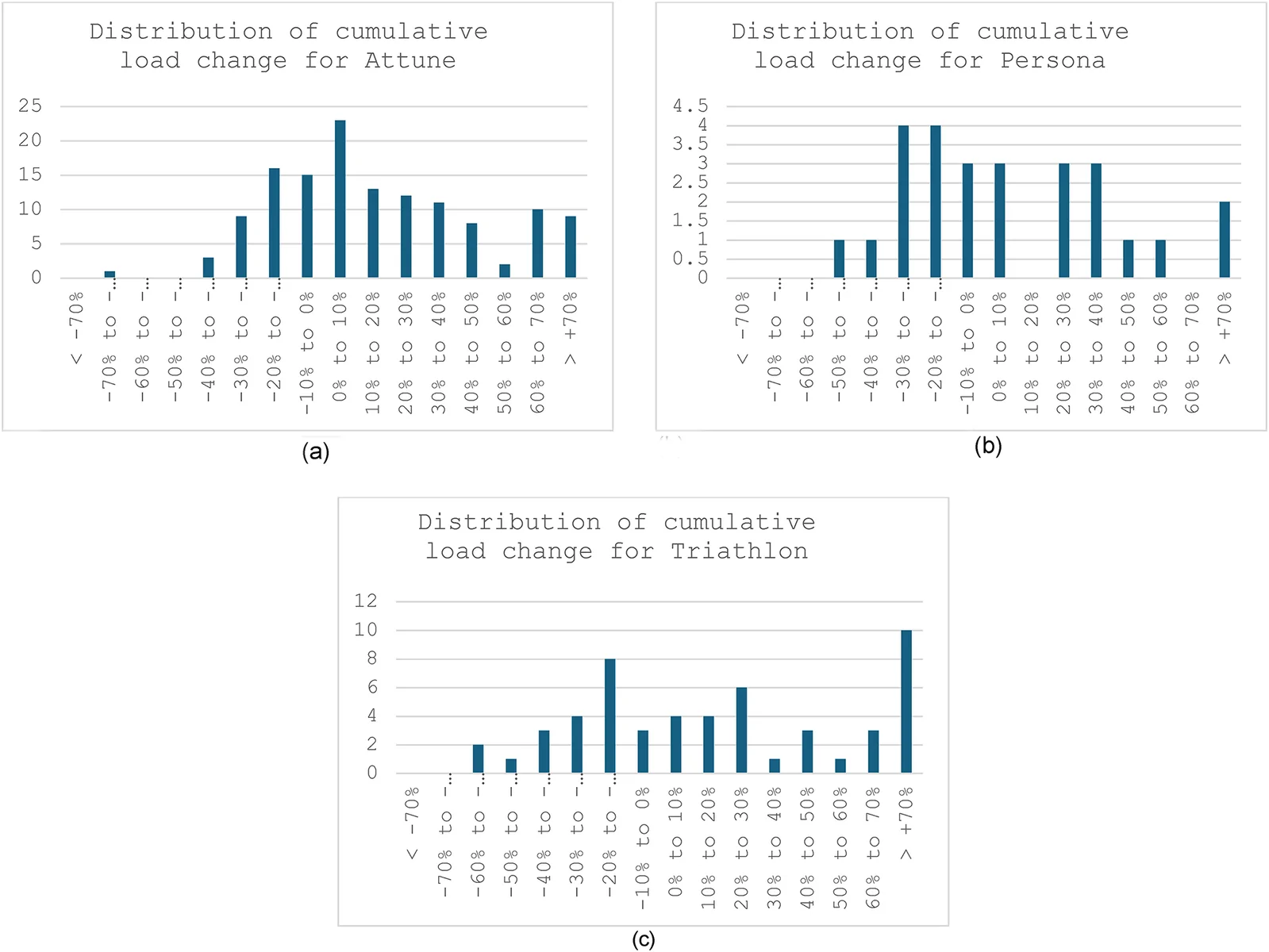
Distribution of percentage cumulative patellofemoral joint load change following TKR procedure using **a** Attune (n = 132), **b** Persona (n = 26), and**c** Triathlon (n = 53). 10% bin widths are used, with outer bins of <-70% and >+70%, corresponding to the outer limits derived from the upper decile of the all cases cohort (n = 291)

 As shown in Fig. 4, Attune and Persona implants were observed to demonstrate broadly similar distributions, characterised by clustering around 0% change and a moderate proportion of cases exhibiting large cumulative load increases. In contrast, the Triathlon implant was associated with a descriptively different distribution, with a substantially higher observed frequency of cases falling within the > + 70% cumulative load increase bin. The Triathlon cohort showed greater variability across the lower and mid-range percentage change bins compared with Attune and Persona.

### Magnitude of compartment-specific load change

**Fig. 5:**
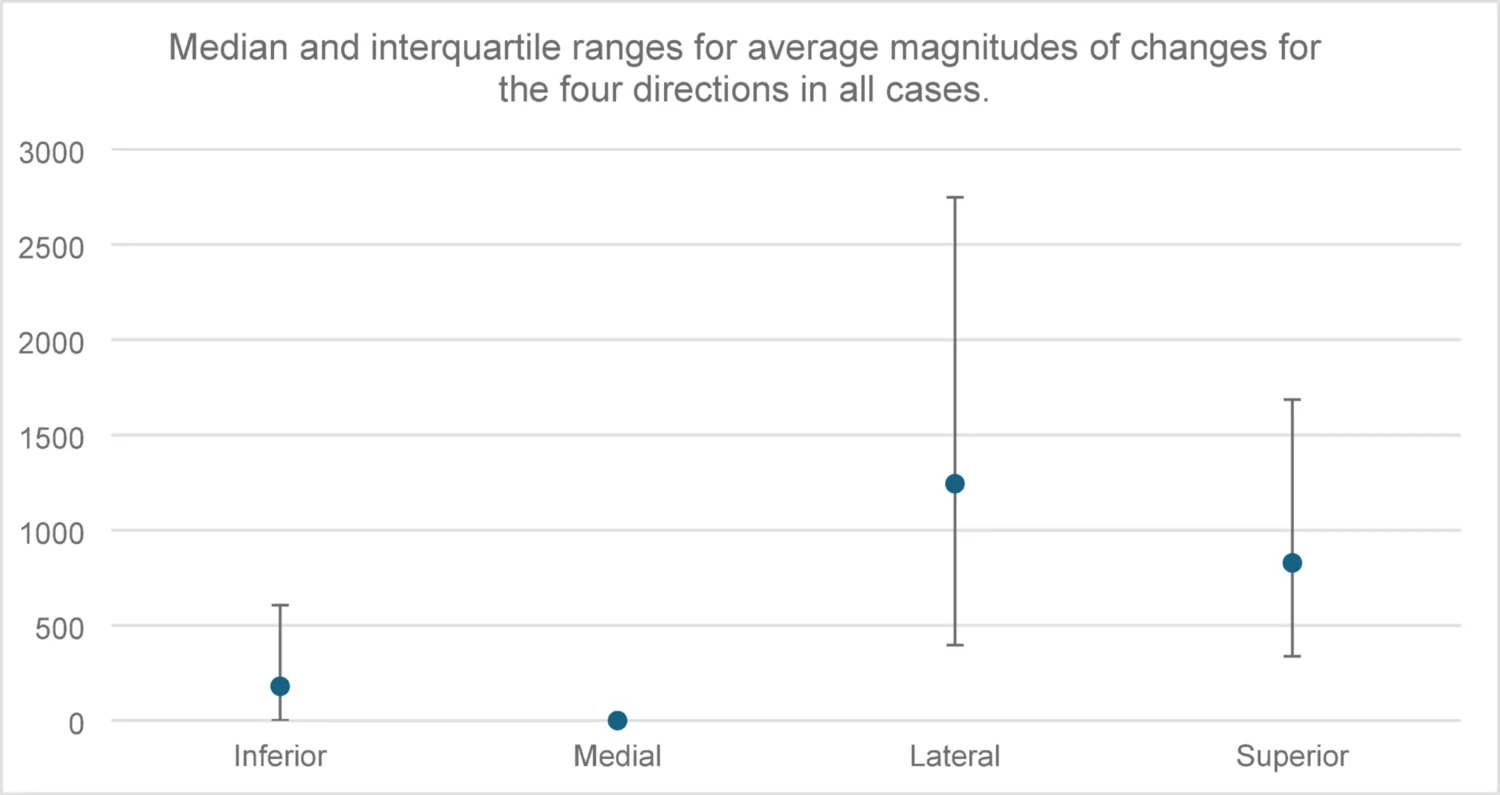
Median and IQRs for average modulus values of magnitude of compartmental load change (*n* = 291). Each individual surgery’s compartmental load values are derived from the mean values of the peak maxima derived from at least three consistent flexion-extension cycles of the supine patient’s leg

Analysis of the modulus of absolute directional load change demonstrated that lateral and superior compartments accounted for a greater proportion of overall observed load change magnitude within the dataset (Fig. 5).

Median modulus values and interquartile ranges demonstrated that medial compartment load remained unchanged in most cases, with both the median and interquartile range centred at zero. Inferior compartment changes were similarly limited. In contrast, lateral and superior compartments demonstrated larger magnitude changes, with higher median values and wider interquartile ranges.

### Directional tendency of compartmental load change

**Fig. 6:**
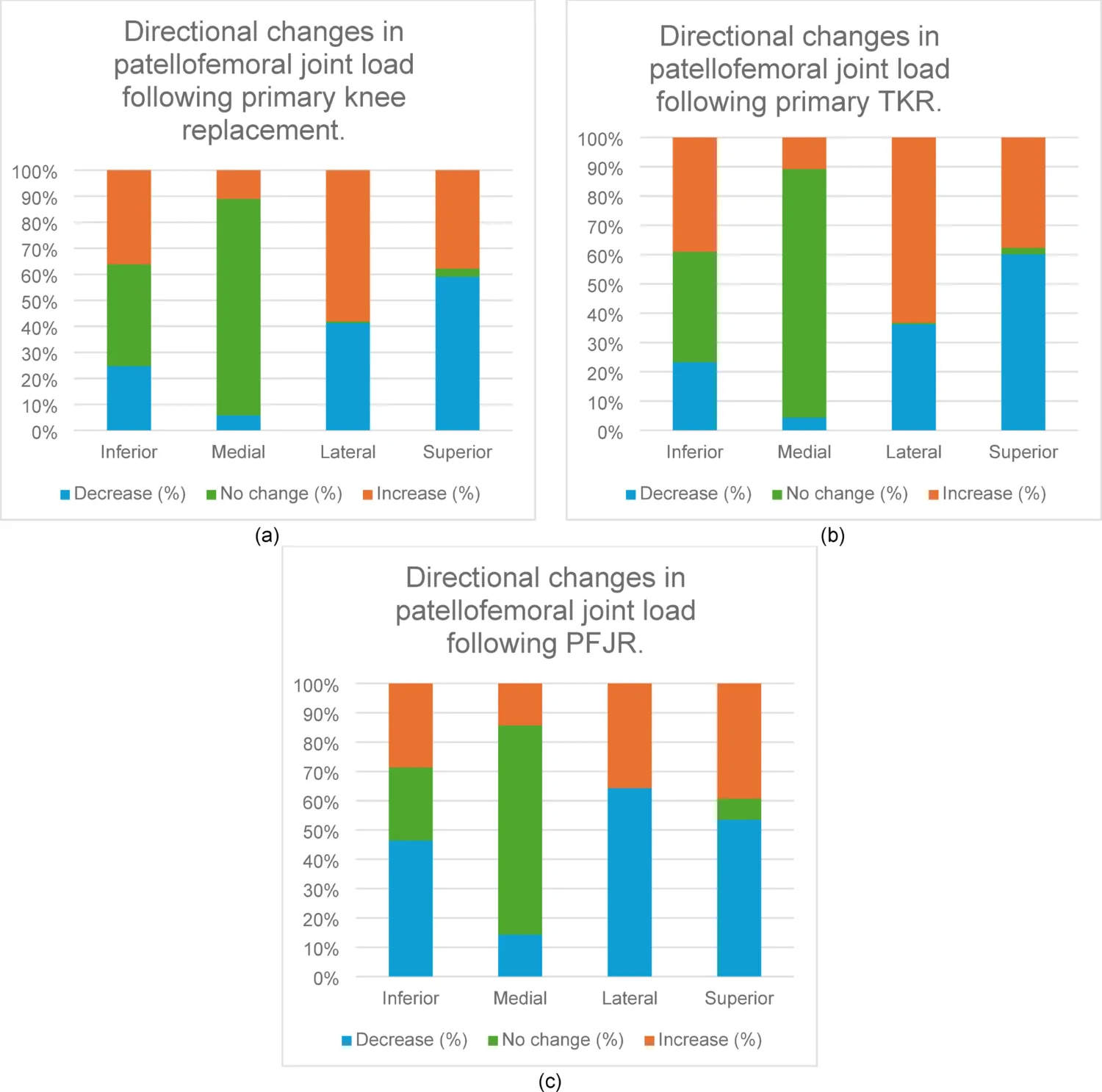
Directional change tendency for compartment loads for **a** all cases (n = 291), **b** TKR (n = 223), and **c** PFJR (n = 28)

Figure 6 summarises the directional tendency of compartmental patellofemoral load change across all cases.

Lateral and superior compartments most frequently exhibited changes following implantation, with the majority of cases demonstrating either an increase or a decrease in load between native and trial measurements. In contrast, medial and inferior compartments remained unchanged in most procedures, with a high proportion of cases demonstrating no change. Exact equality between native and trial values (Δ = 0) was uncommon, reflecting the sensitivity of the measurement system. This analysis therefore focuses on the direction of change rather than the presence or absence of minimal variation. Lateral load increases were more common than decreases in TKR, whereas superior load changes more frequently reflected reductions, consistent with the directional behaviour observed in cumulative and magnitude-based analyses.

### Directional contribution to cumulative PFJ load change

Directional contribution analyses demonstrated that cumulative PFJ load change was predominantly associated with lateral and superior compartments, with medial and inferior contributions being small in comparison.

**Fig. 7:**
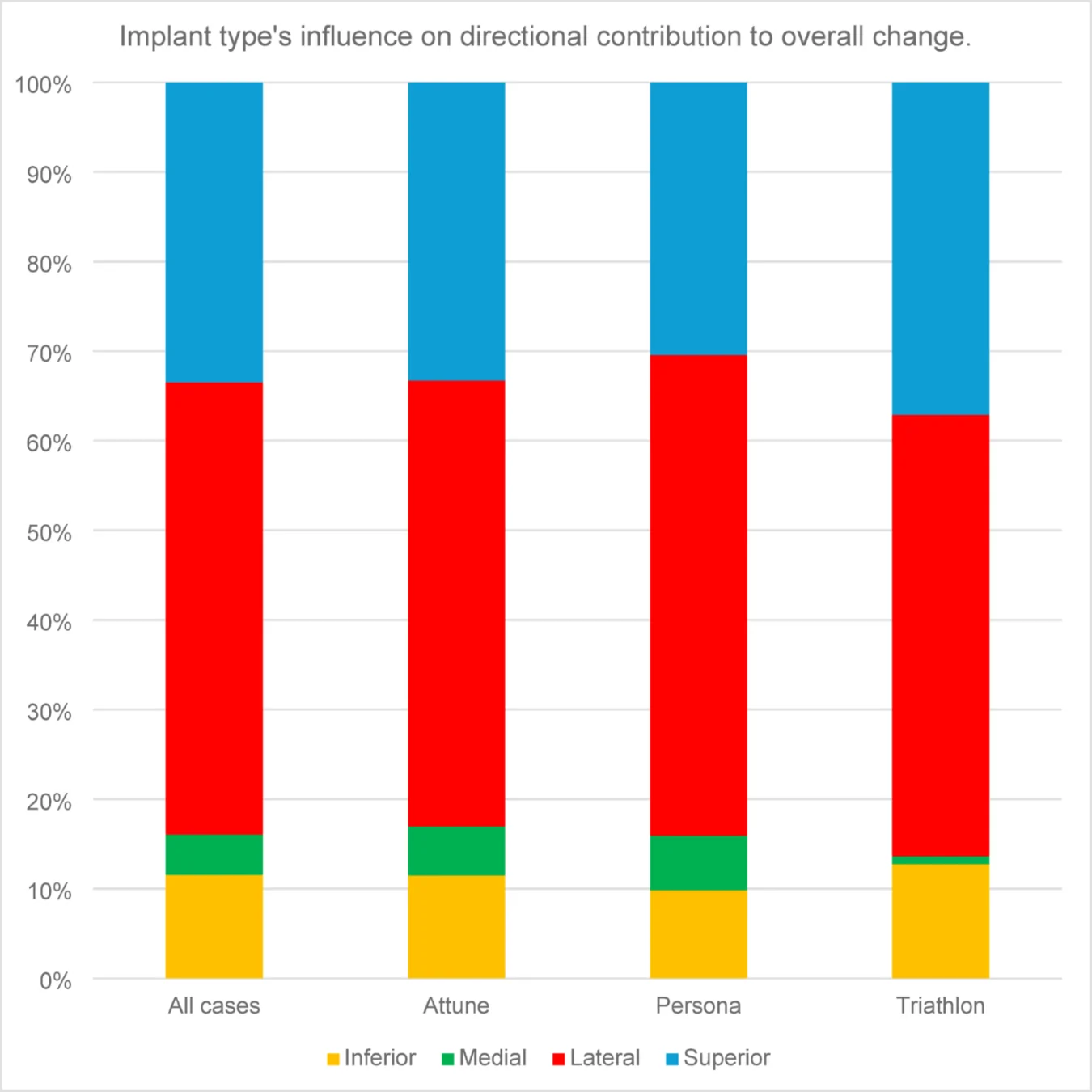
Contribution of total modulus changes by each compartment for implant comparison. For all cases (*n* = 291), and for TKR cases using Attune (*n* = 132), Persona (*n* = 26), Triathlon (*n* = 53)

Figure 7 shows the proportional contribution of each anatomical direction to the total absolute modulus of load change for each implant cohort. Across all implants, a consistent pattern was observed. Lateral compartment changes contributed the largest proportion of overall load redistribution, followed by superior, inferior, and medial compartments. No implant cohort demonstrated a marked deviation from this pattern.

## Discussion

Across this registry cohort, cumulative patellofemoral joint load change demonstrated substantial inter-individual variability. While most cases clustered around relatively small changes in cumulative load, a distinct subgroup exhibited large cumulative increases exceeding + 70%, corresponding to approximately the upper decile of observed changes. This broad distribution around baseline, with a notable tail of large positive changes, indicates that substantial increases in patellofemoral load occur in a meaningful minority of cases rather than representing isolated outliers.

When stratified by procedure type, patellofemoral joint resurfacing procedures demonstrated a descriptively narrower distribution of cumulative load change compared with total knee replacement. PFJR cases clustered more tightly around small changes, whereas TKR procedures exhibited a broader distribution with a higher observed frequency of substantial cumulative load increases. While this pattern may plausibly reflect the greater structural modification inherent to TKR compared with PFJR, the observational design and absence of adjustment for surgical or patient-level variables preclude attribution of these differences to procedure type alone.

Descriptive differences in cumulative load distribution were also observed between implant cohorts within TKR procedures. Attune and Persona implants demonstrated broadly similar patterns characterised by clustering around small cumulative changes with a moderate proportion of larger increases. In contrast, the Triathlon implant cohort demonstrated a higher observed frequency of large cumulative load increases within this dataset. Given that implant use was strongly clustered by surgeon and site, and that patient-level covariates were not available for adjustment, these observations should be interpreted as exploratory and hypothesis-generating rather than as evidence of implant-specific causal effects.

Compartmental analyses demonstrated clear directional patterns in patellofemoral load redistribution. Magnitude-based analysis showed that lateral and superior compartments accounted for the majority of observed load change, whereas medial and inferior compartments exhibited comparatively small changes. Directional tendency analysis further demonstrated that lateral loads most frequently increased following implantation, while superior loads more commonly decreased. Taken together, these findings indicate that cumulative patellofemoral load change within this cohort was primarily driven by redistribution within the lateral and superior compartments rather than uniform changes across the patellofemoral joint.

Several biomechanical factors may plausibly contribute to the observed dominance of lateral and superior compartments. The patellofemoral joint exhibits asymmetric contact mechanics, with the lateral patellar facet typically bearing greater loads than the medial facet due to the quadriceps vector and native patellar tracking tendencies. In the present cohort, the lateral compartment demonstrated both the highest baseline loads and the largest contribution to cumulative load redistribution, accounting for approximately half of the total modulus of load change. Lateral load increased in approximately 58% of procedures and contributed on average around 50% of total load redistribution, indicating that this compartment was the dominant driver of cumulative load change across the cohort.

In contrast, superior compartment loads were more frequently observed to decrease following implantation, occurring in approximately 59% of procedures and contributing roughly one third of total load redistribution. Superior patellofemoral contact forces are influenced by quadriceps tension and the proximal contact region of the patella during knee flexion. Changes in trochlear articulation, restoration of patellar thickness, or alterations in extensor mechanism tension following component implantation may therefore shift contact forces distally or laterally across the patella, resulting in the observed reduction in superior load. The concurrent increase in lateral loading and reduction in superior loading may therefore reflect redistribution of patellofemoral contact forces rather than uniform increases in overall joint loading.

From a clinical perspective, the observed variability in intraoperative patellofemoral load redistribution highlights the potential value of real-time load measurement during knee replacement. Identification of procedures demonstrating large cumulative load increases may allow surgeons to consider intraoperative adjustments such as modification of component rotation, soft-tissue balancing, or patellar preparation. While the present study does not establish a direct relationship between load redistribution and postoperative symptoms, the ability to identify high-load patterns intraoperatively may provide a framework for future investigations evaluating whether targeted adjustments could mitigate patellofemoral complications.

Strengths of this study include the prospective collection of intraoperative patellofemoral load data across a large, multi-centre cohort, the use of a validated load-sensing system, and the ability to interrogate both cumulative and compartment-specific load behaviour. The use of frequency-based analyses allows visualisation of variability and identification of clinically relevant subgroups that may not be apparent when considering mean values alone.

This study has several limitations. As an observational registry analysis, surgical variables such as implant choice and operative technique could not be fully isolated, and some overlap between cohorts was unavoidable. Implant selection was not randomised and was frequently clustered by surgeon and centre, and no multivariable adjustment for potential confounding factors, including patient characteristics, surgical technique, or centre-level variables, was performed. In addition, clinical outcomes were not assessed, and no causal relationship between patellofemoral load redistribution and postoperative symptoms can be inferred from these data. Further studies incorporating standardised surgical protocols and outcome measures would be required to determine the clinical relevance of the load changes observed.

## Data Availability

The datasets generated and analysed during the current study are not publicly available due to the proprietary nature of the registry data but may be available from the corresponding author upon reasonable request.

